# Randomized, comparative study of the efficacy and safety of artesunate plus amodiaquine, administered as a single daily intake versus two daily intakes in the treatment of uncomplicated falciparum malaria

**DOI:** 10.1186/1475-2875-7-16

**Published:** 2008-01-19

**Authors:** Jean Louis A Ndiaye, Babacar Faye, Amadou M Diouf, Thomas Kuété, Moustapha Cisse, Papa A Seck, Phillipe Brasseur, Albert Same-Ekobo, Valerie Lameyre, Oumar Gaye

**Affiliations:** 1 Department of Parasitology, Faculty of Medicine, Cheikh Anta Diop University, Dakar, Senegal; 2 Department of Parasitology, Faculty of Medicine Yaounde, Cameroon; 3 Ministry of Health and Medical Prevention, Sénégal; 4 Research and Development Institute, Dakar, Sénégal; 5 Impact Malaria, Sanofi-aventis, Paris, France

## Abstract

**Background:**

Artesunate plus amodiaquine is a coblistered ACT, given as a single daily intake. It has been suggested that, in view of the number of tablets to be taken (particularly in adults), it may be possible to improve compliance by allowing patients to divide the daily dose. The objectives of this randomized, comparative, open-label, multicentre study, conducted in Senegal and in Cameroon in 2005, was to demonstrate the non-inferiority and to compare the safety of artesunate plus amodiaquine, as a single daily intake versus two daily intakes.

**Methods:**

A three-day treatment period and 14-day follow-up period was performed in any subject weighting more than 10 kg, presenting with a malaria paroxysm confirmed by parasitaemia ≥ 1,000/μl, after informed consent. Patients were randomly allocated into one of the two regimens, with dosage according to bodyweight range. All products were administered by an authorized person, blinded to both the investigating physician and the biologist. The primary endpoint was an adequate response to treatment on D14 (WHO definition). The two-sided 90% confidence interval of the difference was calculated on intent to treat (ITT) population; the acceptance limit for non-inferiority was 3%. The safety was evaluated by incidence of adverse events.

**Results:**

Three-hundred and sixteen patients were included in the study. The two patient groups were strictly comparable on D0. The adequate responses to treatment were similar for the two treatment regimens on D14, PCR-corrected (99,4% in the one-daily intake group versus 99,3% in the comparative group). The statistical analyses demonstrated the non-inferiority of administering artesunate/amodiaquine as two intakes. The drug was well tolerated. The main adverse events were gastrointestinal disorders (2.5%) and pruritus (2.5%); safety profiles were similar in the two groups.

**Conclusion:**

This pilot study confirms the efficacy and good tolerability of artesunate plus amodiaquine, administrated either in one or in two daily intakes.

## Background

Nearly all currently available antimalarial monotherapies are facing resistance, the severity and incidence of which may vary according to endemic areas. Only one class of antimalarials, artemisinin derivatives and artesunate in particular, has been able to escape this phenomenon so far, most probably owing to its recent introduction, its mechanism of action and its rapid elimination from the body. National and international experts, in agreement with the WHO [1], have proposed a treatment option combining antimalarial agents with a prolonged schizonticidal effect with an oral artemisinin derivative for the treatment of uncomplicated *Plasmodium falciparum *attacks.

The onset of resistance to this type of treatment combination is unlikely, partly because the combined agents have different mechanisms of action, and also because the artemisinin derivative very rapidly reduces the parasite biomass which enables lasting exposure of the few remaining parasites to a high concentration of the combined drug.

A number of controlled phase III studies under the auspices of the TDR [2], together with a number of large-scale studies [3] have investigated bitherapy with artesunate combined with amodiaquine. This well-tolerated combination in no way increases the possible undesirable effects arising from amodiaquine alone, but improves the cure rates in countries where the latter antimalarial has become less effective. Phase IV studies, conducted in Senegal, the Comoros and Mali in over 1,000 patients, have confirmed its efficacy in the region of 95 to 99%, and its good clinical and biological safety.

Bitherapy with artesunate and amodiaquine has obtained a marketing authorization in several African countries, including Senegal and Cameroon, under the Arsucam^® ^trademark.

Artesunate plus amodiaquine was evaluated as a single daily intake in all of the studies already performed. The number of tablets to be taken for each intake (particularly in adults: eight tablets per intake) suggests that compliance may be improved by allowing patients to divide tablet intake into two daily intakes. The objective of this multicentre, randomized, open-label phase IV study, was to demonstrate the non-inferiority, in terms of clinical and parasitological efficacy on D14, of the administration of artesunate/amodiaquine as a single daily intake versus two daily intakes. The safety and tolerability of treatment were also studied.

## Methods

### Study area and population

The study took place in three sites, two in south Senegal, where the climate is soudanian and the rainy season concentrated between June and October and one site in central Cameroon with a sub-equatorial climate, the rainy season is from April to November.

Any adults or children weighing ≥ 10 kg, able to receive oral treatment, suffering from uncomplicated malaria attack with parasitaemia ranging from 1,000 to 200,000/μl (moderate and high transmission area) and axillary temperature ≥ 37.5°C or history of fever within the previous 24 hours, was enrolled after signed informed consent. Patients could not be included in the study if at least one sign of severe malaria or clinical danger (WHO definition) was present: serious concomitant disease, allergy to one of the investigational medicinal products, pregnancy, or documented intake of an antimalarial at a suitable dosage within seven days prior to inclusion.

An ethical approval was given by the *Conseil National de Recherche en Santé *in Senegal and the *Comité National d'Ethique *in Cameroon on 17 June 2005 and 23 May 2005, respectively.

### Determination of sample size

Based on previous studies, with the hypotheses P_1 _= 99% as the envisaged response rate for artesunate plus amodiaquine single dose (reference), a non-inferiority limit of 3%, a type I (α) error probability of 5% and a type II (β) error probability of 20%, the number of patients per group corresponded to 136. In order to anticipate premature withdrawals, a decision was made to include 150 patients per group, i.e. a total of 300 patients. Each site was, therefore, required to recruit 100 patients. In order to avoid any imbalance between the different weight categories, the randomization list was drawn up by centre and by weight range.

### Intervention

During the inclusion visit (D0), a physical examination was performed with recording of medical history, demographic data (age, gender), weight and height, and measurement of vital signs (blood pressure, pulse in the supine position), and axillary's temperature, together with evaluation of symptoms. Parasitological tests (blood films, gametocytaemia) together with PCR filter paper were also performed.

Follow-up visits took place on D1, D2, D3, D7 and D14 during which a physical examination was performed, with evaluation of clinical safety and symptoms, measurement of vital signs, and parasitological tests (except on D1 and D2). Filter paper blood spot samples were prepared on D14, if the thick blood film result was positive, with a view to PCR analysis.

Since the artesunate plus amodiaquine coblisters contain one tablet of 50 mg artesunate and one tablet of 153 mg amodiaquine, dosages had to be adapted to the patient weight range: patients weighing between 10 and 21 kg received one tablet of each product per day, patients weighing ≥ 21 and ≤ 40 kg received two tablets of each product per day and patients weighing >40 kg received 4 tablets of each product per day. According to randomization, tablets were given either once or twice a day. The duration of treatment was three days in the two groups.

Each patient was randomly allocated to one of the study groups, according to a pre-defined randomization list. The tablets were administered with a small amount of water. For the younger children, the tablets were crushed and administered with water. All treatments were administered in the presence of nursing staff. The patients stayed at the centre for 30 minutes after administration, for observation. If the patient rejected the treatment during the observation period, the same dose was re-administered. In the event of repeated vomiting, the patient had to be withdrawn from the study and a replacement treatment had to be introduced. All possible steps were implemented in order to avoid losing patients to follow-up: active follow-up, even going as far as the patient's home, was carried out by a member of the principal investigator's team.

### Parasitological assessment

Thick films together with thin blood films were prepared at each study visit with the exception of D1. A molecular biology analysis (PCR) was performed on inclusion and on D14 +/- 1d in the event of a positive thick film on D14 +/- 1d. Slide-reading and parasite counts were performed on site and considered as negative in absence of asexual *P. falciparum *form after examination of 300 leucocytes; 10% of the slides were re-read at the centre's reference Parasitology Department at Dakar University.

The molecular biology analyses were performed at the parasitology laboratory at the Faculty of Medicine, Université Cheikh Anta Diop Dakar – Senegal using filter paper blood spot samples prepared in the field. The distinction of reinfections or recrudescence cases was carried out using genetic markers for *msp-1*, *msp-2 *and microsatellites

### Statistical analysis

Independent duplicate data entry was performed using the SAS software program, version 8.2, in compliance with the standards stipulated by GCP guidelines. All of the qualitatives and quantitatives variables recorded were described according to treatment group and globally. The 95% confidence intervals were calculated.

The non-inferiority of artesunate plus amodiaquine as two intakes versus artesunate plus amodiaquine as a single intake was studied on the basis of the adequate response to treatment on D14 (WHO definition). The two-sided 90% confidence interval of the difference in the proportions of responders on D14 between the two treatment groups was calculated. If the upper limit of the confidence interval was below the acceptance limit (d = 3%), the non-inferiority of artesunate plus amodiaquine in two intakes versus a single intake would be demonstrated. This analysis was conducted on the ITT and per protocol (PP) populations. The main analysis corresponded to the ITT population.

The safety of artesunate plus amodiaquine was evaluated by the presence or absence of adverse events, type of adverse event, intensity and the causal relationship with the investigational product in the safety population.

## Results

**Figure 1:**
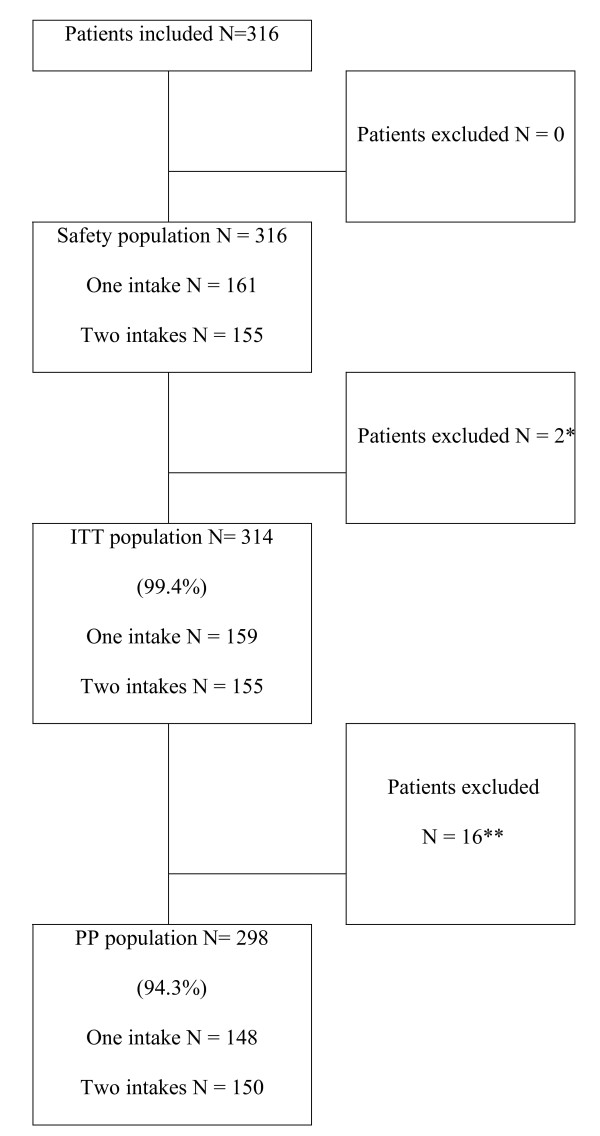
**Study design**. (*) Two patients did not undergo a parasite evaluation between D3 and D14 (patients withdrawn from the study). (**) 11 patients presented major deviations and five patients did not complete the study. Five patients, three in the single-intake group and two in the two-intake group, did not undergo an evaluation on D14. These patients moved out of the area covered by the investigating centre before the end of the study.

From June to October 2005, 316 patients were included in the study. 161 patients were assigned to the artesunate plus amodiaquine single-intake treatment group (reference) and 155 to the artesunate plus amodiaquine two-intake treatment group. Seven patients were withdrawn from the study: five patients in the single-intake group and two in the two-intake groups. Only one patient (in the single-intake group) was withdrawn due to an adverse event, five patients were withdrawn because of loss to follow up and one because of consent withdrawn (Figure 1)

On inclusion, the patients in the two treatment groups presented similar data (Table 1), with a mean body temperature (± standard deviation) of 38.30°C (± 1.01°C), median values of parasitaemia equal to 16,421.0 parasites/μl in the single-intake group versus 13,750.0 parasites/μl in the two-intake group.

**Table 1:** Clinical and parasitological baseline data on D0

	**Artesunate plus amodiaquine One intake/day**	**Artesunate plus amodiaquine Two intakes/day**	**Total**
**Patients**	161	155	316
Male	97 (60.2%)	85 (54.8%)	182 (57.6%)
Female	64 (39.8%)	70 (45.2%)	134 (42.4%)
**Age range**			
≤ 5 years	32 (19.9%)	26 (16.8%)	58 (18.4%)
From 6 to13 years	65 (40.4%)	63 (40.6%)	128 (40.5%)
≥ 14 years	64 (39.7%)	66 (42.6%)	130 (41.1%)
**Parasite density/μL**			
Mean (SD)	41861.3 (75507.9)	31940.6 (42530.8)	36995.2 (61684.5)
Médian (Q1; Q3]	16421(6473; 45550]	13750(6240; 45217]	15423.0 (6356.5;45378.5]
**Gametocytes/μL**			
Mean (SD)	21.9 (134.5)	18.8 (110.9)	20.4 (123.3)

### Efficacy outcome

The primary efficacy endpoint was the adequate response to treatment after PCR analysis (ACPR_c_). Irrespective of treatment regimen, more than 99% of patients showed an adequate clinical and parasitological response.

The results of the evaluation of efficacy on D14 before PCR and after PCR on the ITT population are presented on Table 2. The two-sided 90% confidence interval of the difference in the proportion of PCR corrected adequate clinical and parasitological response (ACPR_c_) between the two treatment groups was: (-3.00; 1.11]. The upper limit of the confidence interval (1.11%) was below the acceptance limit, d = 3%., assessing that the two intakes treatment regimen is non-inferior to the single intake treatment regimen on the ITT population. These results were confirmed on the PP population.

**Table 2:** Evaluation of efficacy on D14 before PCR correction (ITT population)

	**Artesunate plus amodiaquine one intake**	**Artesunate plus amodiaquine two intakes**	**Total**
ETF (Early treatment failure)	0	0	0
LCF (Late clinical failure)	0	0	0
LPF (Late parasitological failure)	1 (0.6%)	1 (0.7%)	2 (0.6%)
ACPR (Adequate clinical and parasitological response)	155 (99.4%)	152 (99.3%)	307 (99.4%)
ACPR_c _(Adequate clinical and parasitological response after PCR correction)	155 (99.4%)	153 (100.0%)	308 (99.7%)
			
NA	3	2	5

Note: "NA" = "Missing" patient not having undergone parasitological tests on D14.

**Figure 2:**
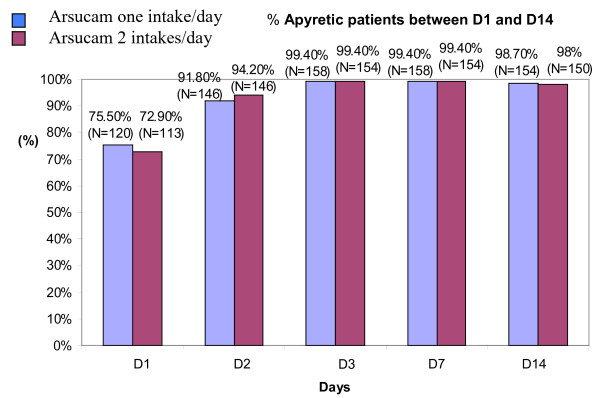
Apyretic patients between D1 and D14.

The changes in mean temperature during the study were similar in the two treatment groups, with values for mean temperature below 37.5°C as from D1. The percentage of apyretic patients between D1 and D14 is shown in Figure 2. Between D2 and D14, the number of patients with negative parasitaemia increased in a similar manner in the two treatment groups: from approximately 70% on D2 to more than 99% on D14.

Gametocyte-carrier patients were 12 on inclusion, D7 and D14, and 14 on D3. The mean values for gametocytaemia were halved between D1 and D7 in both groups. With the exception of anorexia wich mainly affected patients under seven years of age in the two treatment groups, and was on D1 statistically higher in the single-intake group (37.9% versus 28.3%, p = 0.0389). A similar and favourable outcome, with regard to the symptoms of malaria, was observed for the two patient groups during the study. The changes in the percentage of patients in each group presenting these symptoms are shown in the Table 3.

**Table 3:** Changes in the percentage of patients presenting symptoms of malaria attack during the study

Symptoms	Percentage of patients with symptoms on D0		Percentage of patients with symptoms on D3		Percentage of patients with symptoms on D14	
	
	One intake N = 161	Two intakes N = 155	One intake N = 161	Two intakes N = 155	One intake N = 161	Two intakes N = 155
Headache	88.8%	91.0%	5.0%	1.9%	0.6%	0.7%
Perspiration	59.0%	55.5%	10.6%	9.7%	1.2%	3.9%
Dizziness	42.2%	45.2%	8.7%	7.1%	0.6%	1.3%
Chills	69.6%	67.7%	3.1%	4.5%	0.6%	2.6%
Pain	41.6%	40.6%	5.7%	10.3%	1.9%	2.6%
Anorexia	70.8%	71.6%	19.8%	15.5%	1.2%	0
Asthenia	77.0%	74.8%	39.1%	38.7%	0.6%	0.6%
Jaundice	1.9%	0.6%	0.6%	0	0	0
Skinfold	0.6%	0.6%	1.3%	1.3%	0	0
Hepatomegaly	7.5%	5.8%	7.5%	3.9%	5.8%	3.9%
Vomiting in 24 hours	47.8%	43.9%	3.7%	2.5%	0	0
Diarrhoea in 24 hours	9.3%	8.4%	1.8%	2.5%	0	1.3%

### Safety outcome

Certain symptoms attributable to malaria, but not present on inclusion, appeared during treatment: asthenia (5.7%), vomiting (4.7%), anorexia (4.7%), diarrhoea (2.8%), abdominal pain (1.9%) and dizziness (2.2%), without any differences between the treatment groups. The influence of the number of tablets per intake, or the total quantity of drug substance to be ingested, on the onset of these symptoms was the subject of a descriptive analysis only, owing to the limited sample size.

With the exception of two patients prematurely withdrawn from the study in the single-intake group (one for consent withdrawn, the other for bronchopneumopathy), who only received treatment for one day, all of the other patients received treatment for three days, as provided for in the protocol. The adverse events (AE) recorded during the study were coded in compliance with the MedDRA dictionary, and described for the safety population.

Among the population included, the AEs reported during the study mainly corresponded to gastrointestinal disorders (2.5%) or pruritus (2.5%), irrespective of the treatment regimen. Three patients in the two-intake group reported AEs related to nervous system disorders (two cases of drowsiness and one case of dysgeusia – bitter taste) versus one patient in the single-intake group (one case of seizure). All of the gastrointestinal or nervous system disorders and cases of pruritus reported during the study were perceived as related to the investigational product, irrespective of treatment regimen. Intensity of adverse events reported as being related to the investigational product is shown in Table 4.

**Table 4:** Intensity of adverse events reported as being related to the investigational product

	**One intake N = 161**			**Two intakes N = 155**		
	
**AE intensity/type**	Mild	Moderate	Severe	Mild	Moderate	Severe
**Gastrointestinal**	1	4	0	2	1	0
**Abdominal pain**	0	2	0	0	1	0
**Duodenal ulcer**	0	1	0	0	0	0
**Nausea**	1	1	0	2	0	0
**Nervous system**	0	0	1	3	0	0
**Dysgeusia**	0	0	0	1	0	0
**Drowsiness**	0	0	0	2	0	0
**Seizures**	0	0	1	0	0	0
**Skin**	4	1	0	2	1	0
**Pruritus**	3	0	0	0	1	0
**Generalized pruritus**	1	1	0	2	0	0

**Total**	5	5	1	7	2	0

Two serious adverse events occurred during the study, one in each treatment regimen; they were both perceived as unrelated to the investigational product. No death occurred during this study.

Rejection of treatment during the first half-hour after administration was observed in 10 patients, six in the artesunate plus amodiaquine single-intake group and four in the two-intake group, mainly occurring on D0 (Table 5). Out of these 10 patients, seven were aged between six and 13 years. No differences were observed between the two patient groups. An equivalent dose of treatment was re-administered in each case. It should be noted that all patients presented episodes of vomiting in the 24 hours prior to inclusion. The influence, on early treatment rejection, of the number of tablets to be ingested was the subject of a descriptive analysis only owing to the limited sample size.

**Table 5:** Number of patients having experienced early treatment rejection during the study

Number of patients with episodes of vomiting within 30 minutes/Number of tablets per day	One intake N = 161			One intakes N = 155			Total N = 316
		
	2	4	8	2	4	8	
D0	1	1	0	1	3	0	6
D1	0	2	0	0	0	0	2
D2	0	0	2	0	0	0	2

Total	1	3	2	1	3	0	10

Fifteen patients presented episodes of vomiting during the treatment period although this symptom was not observed on inclusion: seven patients (4.3%) in the single-intake group and eight patients (5.2%) in the two-intake group. It should be noted that this description does not take early treatment rejection into account. All these patients presented at least one of vomiting in the previous 24 hours. The number of tablets taken by the 15 patients concerned is shown in the Table 6.

**Table 6:** Number of tablets taken by patients with episodes of vomiting during treatment only

Number of tablets per day	One intake N = 161	Two intakes N = 155	Total N = 316
2 tablets	0	0	0
4 tablets	1 (0.6%)	4 (2.6%)	5 (1.6%)
8 tablets	6 (3.7%)	4 (2.6%)	10 (3.2%)

Total	7 (4.3%)	8 (5.2%)	15 (4.8%)

The episodes of vomiting during treatment appear to have been influenced by the number of tablets taken each day. Dose fractionation does not appear to have any impact on the number of patients having presented this symptom during treatment. However, no conclusions may be drawn owning to the limited sample size in these subgroups.

## Discussion

The correct prescription of ACT by healthcare staff and the compliance of patients are essential for an effective management of malaria in Africa. Different previous studies had already shown the efficacy and tolerability of artesunate plus amodiaquine in sub-Saharan Africa [2-7].

The objective of this pilot study was to verify the non-inferiority of two daily intakes versus a single intake (which can permit to decrease the number of tablet to be taken per dose) and to evaluate the impact of dose fractionation on clinical safety

This study showed that adequate responses to treatment were similar for the two treatment regimens, and approaching 100% before and after PCR analysis on D14. The statistical analyses conducted on the ITT and PP populations demonstrated the non-inferiority of administering Artesunate plus amodiaquine as two intakes versus a single daily intake, in terms of clinical and parasitological efficacy on D14.

It was also demonstrated that the two tested treatment regimen were well tolerated; the observed AEs were similar in terms of type and incidence in the two treatment groups. No changes were observed in the safety profile when dividing Artesunate plus amodiaquine treatment into two daily intakes. The events reported during the study were expected and have already been described with this type of antimalarial.

Numerous papers described an impact of artemisinin derivatives on gametocyte carriage, by killing immature stages of gametocytes. During this study, the number of gametocyte-carrier patients remained stable during follow-up (from D0 to D14), but the mean values for gametocytaemia were halved between D1 and D7 in both groups. This can possibly be explained by the presence of different stage of gametocytes in the study populations.

The duration of follow-up of this study was only 14 days, but the efficacy has been demonstrated in numerous other studies within a D28 follow-up [2,4,5], and the impact of dose fractionation should be observed only in the first days after treatment. Since one of the objectives of the study was to evaluate the impact of taking a large number of tablets in one go on tolerability, no placebo was used during the trial, and a double-blind double-dummy scheme was not used, but treatment administration was blinded for the investigators.

## Conclusion

The combination artesunate plus amodiaquine has already been adopted as first line treatment of uncomplicated malaria attack in 18 countries in sub-Saharan Africa, with a regimen of one daily intake for three days. Even if the tolerability profile remains good in the clinical trials, in real life, some patients (mainly adults) complain about the too important number of tablets to be ingested at once. The results of this study will permit to divide the daily intake into two administrations.

A fixed dose combination of these two active components has already been developed and is currently being evaluated in sub-Saharan Africa. This new formulation, with a limited number of tablets per intake may allow a better adherence to an effective treatment for African communities.

## Competing interests

The author(s) declare that they have no competing interests.

## Authors' contributions

JLAN: designed the study, collected data, and prepared the manuscript

BF : collected data and contributed in the preparation of the manuscript

VL: designed the study and prepared the manuscript

Ph B and ASE : participated in the design of the study and collected data

AMD, PAS, M C and T K ; collected data on field

OG : designed the study and prepared the manuscript
